# Neuromuscular Disorders in Children Through the Lens of Next-Generation Sequencing: A Study of Diagnostic Yield

**DOI:** 10.3390/ijms27093949

**Published:** 2026-04-29

**Authors:** Slavica Ostojić, Gordana Kovačević, Nikola Ilić, Nina Marić, Marina Anđelković, Tanja Lalić, Marijana Mišković, Kristel Klaassen Ljubičić, Irena Marjanović, Aleksandra Paripović, Mihail Baša, Vladislav Vukomanović, Jovana Krstić, Milica Adamović, Aleksandar Sovtić, Adrijan Sarajlija

**Affiliations:** 1 Department of Neurology, Mother and Child Health Care Institute of Serbia “Dr Vukan Čupić”, 11070 Belgrade, Serbia; gordana.kovacevic@imd.org.rs; 2 Faculty of Medicine, University of Belgrade, 11070 Belgrade, Serbia; vladislav.vukomanovic@imd.org.rs (V.V.); aleksandar.sovtic@imd.org.rs (A.S.); adrijan.sarajlija@imd.org.rs (A.S.); 3 Division of Clinical Genetics, Mother and Child Health Care Institute of Serbia “Dr Vukan Čupić”, 11070 Belgrade, Serbia; ilicnikola91@gmail.com; 4 Clinic for Children Diseases, University Clinical Center of the Republic of Srpska, 78000 Banja Luka, Bosnia and Herzegovina; ninamaric.bl@gmail.com; 5 Medical Faculty, University of Banja Luka, 78000 Banja Luka, Bosnia and Herzegovina; 6 Institute of Molecular Genetics and Genetic Engineering, University of Belgrade, 11070 Belgrade, Serbia; marina.andjelkovic90@gmail.com (M.A.); kristel.klaassen@imgge.bg.ac.rs (K.K.L.); irena.marjanovic@imgge.bg.ac.rs (I.M.); 7 Laboratory of Medical Genetic, Mother and Child Health Care Institute of Serbia “Dr Vukan Čupić”, 11070 Belgrade, Serbia; tanja.lalic68@gmail.com (T.L.); marijanamiskovic10@gmail.com (M.M.); 8 Department of Nephrology, Mother and Child Health Care Institute of Serbia “Dr Vukan Čupić”, 11070 Belgrade, Serbia; aleksandra.paripovic@imd.org.rs; 9 Department of Pulmology, Mother and Child Health Care Institute of Serbia “Dr Vukan Čupić”, 11070 Belgrade, Serbia; mihail.basa@imd.org.rs; 10 Department of Cardiology, Mother and Child Health Care Institute of Serbia “Dr Vukan Čupić”, 11070 Belgrade, Serbia; 11 Pediatric Clinic, Mother and Child Health Care Institute of Serbia “Dr Vukan Čupić”, 11070 Belgrade, Serbia; jovanakrst98@gmail.com (J.K.); milica.adamovic19@gmail.com (M.A.)

**Keywords:** neuromuscular disorders, next-generation sequencing (NGS), diagnostic yield

## Abstract

Pediatric-onset neuromuscular diseases (NMDs) represent a clinically and genetically heterogeneous group of rare disorders, often posing significant diagnostic challenges due to overlapping phenotypes. Next-generation sequencing (NGS), particularly whole-exome sequencing (WES), has transformed the diagnostic landscape; however, its clinical utility varies across phenotypic subgroups. We conducted a combined retrospective–prospective cohort study that included 100 pediatric patients with suspected neuromuscular disorders evaluated at a tertiary referral center between 2015 and 2025. Patients were stratified into seven clinically defined diagnostic categories prior to genetic testing. NGS-based diagnostics (primarily WES) were performed following initial clinical and targeted evaluations. Diagnostic yield and predictors of a positive genetic result were analyzed using univariate and multivariable statistical approaches. A molecular diagnosis was established in 71% of patients, including 64% with pathogenic/likely pathogenic variants and 7% with clinically consistent variants of uncertain significance. Diagnostic yield varied significantly across disease categories (*p* < 0.001), reaching near-complete rates in well-defined phenotypes such as congenital myasthenic syndromes, neuropathies, and metabolic myopathies, while markedly lower yield was observed in unclassified cases (38.2%). Multivariable logistic regression identified disease group as the only independent predictor of diagnostic success (B = −0.436, *p* = 0.001). Frequently implicated genes included *DMD*, *RYR1*, and *LAMA2*, reflecting a predominance of structural and excitation–contraction coupling defects. NGS demonstrates high diagnostic utility in pediatric neuromuscular disorders, particularly when applied in a phenotype-driven framework. Diagnostic performance is strongly influenced by the degree of clinical definition prior to testing, highlighting the continued importance of expert phenotyping in the genomic era.

## 1. Introduction

Pediatric-onset neuromuscular diseases (NMDs) comprise a heterogeneous group of rare and frequently progressive disorders that affect the peripheral nervous system, the neuromuscular junction, and/or skeletal muscle [1]. The core phenotypic features include hypotonia, muscle weakness, delayed motor milestones, and joint contractures, alongside diverse systemic manifestations.

Most NMDs are classified as rare diseases [1]. Recent epidemiological studies from Europe suggest an overall joint prevalence of pediatric NMDs ranging between 37 and 41 per 100,000 children [2,3]. Other population-based studies have reported a broader range, from 21.4 to 63.1 per 100,000, reflecting differences in study populations, methodologies, and diagnostic inclusion criteria [2,3]. Given their phenotypic complexity and substantial clinical overlap among distinct entities, establishing an accurate diagnosis remains a considerable challenge in pediatric neurology.

Conventional diagnostic modalities, including electromyoneurography, biochemical assays, imaging studies, and muscle biopsy, remain essential in clinical practice but are often insufficient to precisely define the underlying etiology [1,4]. The advent of molecular genetic testing, particularly next-generation sequencing (NGS), has substantially enhanced the diagnostic capabilities for elucidating the etiology of neuromuscular disorders [5]. Various sequencing strategies, such as targeted gene panels, whole-exome sequencing (WES), and whole-genome sequencing (WGS), enabled simultaneous analysis of numerous genes associated with both known and potentially novel forms of NMDs [4,5,6].

The establishment of a molecular diagnosis provides multiple clinical and scientific benefits: it enables definitive diagnostic confirmation, guides patient management, informs prognostication and genetic counseling, and increasingly facilitates the implementation of targeted and personalized therapeutic approaches [1,4,5,6]. Furthermore, early and accurate diagnosis may alleviate psychosocial stress for affected families and prevent unnecessary diagnostic or therapeutic interventions [7].

We aimed to objectivize our experience that advances in genetic technologies, particularly NGS, have substantially improved diagnostic resolution in patients with suspected neuromuscular disorders who remained undiagnosed after initial clinical and targeted diagnostic evaluation.

## 2. Results

### 2.1. Demographic and Clinical Characteristics

The final cohort included 100 individuals, with the majority being male (71%). Diagnosis most commonly occurred between 1–3 years (21%) and 6.1–11 years (20%) of age. Disease onset was predominantly early, with 27% of patients presenting at birth and a further 24% within the first year of life (Table 1).

The cohort of 100 patients was distributed among the seven pre-defined diagnostic categories (Table 2).

The largest proportion of the cohort consisted of patients with unclassified neuromuscular diseases (UNDs; *n* = 34, 34%). Among the specific suspected diagnoses, Duchenne/Becker muscular dystrophies (DMD/BMD) were the most frequent (*n* = 19, 19%), while the smallest diagnostic group consisted of children with congenital myasthenic syndromes (CMSs; *n* = 4, 4%).

The majority of patients had no comorbidities (70%), while a relatively small proportions presented with associated cardiovascular (6%), endocrine (5%), respiratory (3%), nephrological (3%), and other neurological conditions (6%).

Electrophysiological studies were heterogeneous and incomplete. Namely, electroneuromyography (EMNG) data were missing in nearly half of the cohort (48%). Among patients with available results, 23% showed myopathic changes, 17% had normal findings, and 12% presented with neuropathic features. Muscle biopsy was rarely performed (12% of patients). Centronuclear myopathy was the most frequent histological pattern (3%), followed by inflammatory myopathy [2] and myopathy with tubular aggregates [2].

Family history was negative in the majority of cases, although a minority reported affected first- and second-degree relatives with similar clinical presentation (14%).

### 2.2. Diagnostic Utility of Exome Sequencing

Genomic testing achieved a high diagnostic yield, providing a molecular diagnosis in 71% of the cohort. Pathogenic or likely pathogenic (P/LP) variants explaining the clinical phenotype were identified in 64% of patients, while an additional 7% harbored variants of uncertain significance (VUSs) that were considered clinically consistent with the suspected neuromuscular disorder. Most of the variants identified were rare or absent in population databases such as gnomAD, with allele frequencies consistently below 0.01, qualifying them as putative disease-causing variants. The cohort demonstrated a heterogeneous variant spectrum dominated by missense variants (48.6%), followed by nonsense (19.6%), frameshift (18.7%), and splice-site variants (5.6%), with additional rare intronic variants (0.9%) and small copy number changes, including five deletions (4.7%) and two duplications (1.9%). A list of all 36 genes harboring causative variants associated with the observed phenotypes in the study cohort is presented in Table 3.

In terms of molecular pathways, dystrophinopathies (DMD and BMD) and laminin α2 deficiency (LAMA2) were primarily driven by complete or partial loss of protein expression, leading to structural instability of the muscle fiber. Variants in RYR1 were mostly missense substitutions clustered within functional domains of the ryanodine receptor, consistent with disrupted calcium handling and excitation–contraction coupling. TTN variants were distributed across multiple exons, underscoring the well-established titin-related mechanisms of sarcomere instability. Taken together, the variant distribution reflects a mixture of loss-of-function mechanisms (frameshift/nonsense/splice) and functional disruption through missense variants in genes critical for sarcolemmal integrity, sarcomere assembly, and neuromuscular junction transmission. Importantly, the majority of variants were ultra-rare; population-level controls provided no evidence of benign polymorphism, and segregation data further supported pathogenicity in familial cases.

### 2.3. Analytical Statistics

The proportion of genetically confirmed cases differed significantly among the seven neuromuscular disease categories (Pearson χ^2^ = 28.74, *p* < 0.001). Given that 50% of cells had expected counts below 5, Fisher’s exact test was also examined and confirmed the association (*p* = 0.001).

In contrast, sex, age of onset, presence of comorbidities, and family history showed no statistically significant relationships to the yield of NGS-based diagnostics.

**Figure 1:**
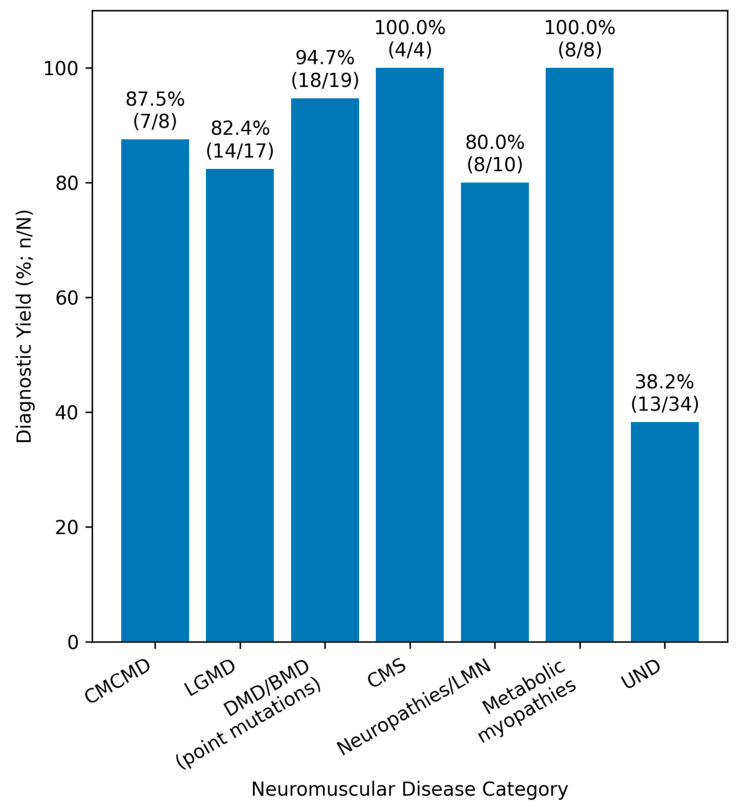
Diagnostic yield of next-generation sequencing across neuromuscular disease categories. **Abbreviations:** CM—congenital myopathies; CMD—congenital muscular dystrophies; LGMD—limb-girdle muscular dystrophies; DMD—Duchenne muscular dystrophy; BMD—Becker muscular dystrophy; CMS—congenital myasthenic syndrome; LMN—lower motor neuron; UND—unclassified neuromuscular disorders, *n*—number in the subgroup, and N—total number of patients in the study.

A multivariable logistic regression model was constructed to evaluate predictors of a positive genetic diagnosis. The overall model was statistically significant (χ^2^ = 22.787, df = 5, *p* < 0.001), explaining a moderate proportion of the variance (Nagelkerke R^2^ = 0.291). Among the tested variables, disease group was identified as the only independent predictor of genetic diagnosis (B = −0.436, *p* = 0.001). Other variables, including sex (*p* = 0.502), age of onset (*p* = 0.485), family history (*p* = 0.500), and presence of comorbidities (*p* = 0.089), were not statistically significant predictors. The diagnostic yield across disease categories is illustrated in Figure 1. The results of the multivariable logistic regression analysis are summarized in Table 4.

Within the undetermined (UND) group, the most common presentation was hyperCKemia, which was observed in 66.0% of patients, followed by psychomotor delay as the leading manifestation in 16.0% of cases, while other clinical presentations accounted for 18.0% (including skeletal abnormalities, multiple congenital anomalies, and other phenotypes). When the analysis was restricted to these defined clinical subgroups, no statistically significant difference in diagnostic yield was observed across categories (χ^2^ = 3.89, df = 2, *p* = 0.14).

## 3. Discussion

In our cohort, exome sequencing (ES) methodologies demonstrated a substantial diagnostic yield in pediatric patients with NMDs, providing an overall diagnostic rate of 71%. Stratified analysis across diagnostic categories revealed the highest yield in well-defined clinical entities, including congenital myopathies/muscular dystrophies, limb-girdle muscular dystrophies, and congenital myasthenic syndromes. Conversely, the lowest proportion of genetically confirmed cases was observed among patients with unclassified suspected NMDs. These findings suggest a high level of accuracy in the initial clinical stratification performed by our specialized neuropediatric team, based on detailed phenotypic assessment and first-line diagnostic investigations.

We acknowledge that EMNG and muscle biopsy data were unavailable for a subset of patients, which may have limited the precision of clinical stratification. This limitation is due to the retrospective nature of a part of the cohort and variability in the availability of diagnostic procedures. During the past decade, limited access to muscle biopsy histopathological analysis represented a practical constraint, contributing to the relatively small number of biopsies performed. Moreover, in pediatric clinical practice, there is a deliberate effort to minimize invasive and potentially painful diagnostic procedures. Consequently, when clinically justified, our diagnostic pathway prioritized comprehensive genetic testing over muscle biopsy, reflecting a contemporary shift toward a genomics-first approach in the evaluation of suspected NMDs.

Multiple relevant studies have reported variable diagnostic yields of NGS-based diagnostic approaches in the evaluation of NMDs, ranging from 19% to 73% [4,8,9]. Tsang et al. reported an overall WES diagnostic yield of 26% in their entire cohort, which varied according to diagnostic subgroup and was comparable to the yield obtained using targeted gene panels (24%). Specifically, the diagnostic rate was 17% among patients with hereditary congenital myopathy and 45% among those within the hereditary muscular dystrophy subgroup [8]. In a heterogeneous cohort of 106 pediatric patients, as reported by Herman et al., the diagnostic yield of clinical ES was 47% [10]. Fattahi et al. documented a consanguinity rate of 52% among the parents of the 45 patients with NMDs included in their cohort, of whom 37 had pediatric-onset disease. In this subgroup of pediatric-onset NMDs, exome sequencing achieved a diagnostic yield of 73% [11]. Interestingly, in the study by Lee et al., whole-genome sequencing demonstrated a diagnostic yield of 43% in pediatric patients with different neurological disorders, which increased to 62.5% among those with neuromuscular diseases [12]. The Slovenian authors Božović et al. reported a notably high diagnostic yield of 64% following WES analysis in a cohort of 22 pediatric patients evaluated for various muscular disorders [13], including DMD, CM, LGMD, and unspecified myopathy. In their cohort, a pathogenic variant was identified in 35% of patients with unclassified myopathy (UM), which is comparable to the detection rate of P/LP variants in our cohort of unclassified NMD patients (31%). Conversely, lower diagnostic yields have been reported in cohorts from higher-resource settings, where WES is often applied as a second-tier diagnostic tool. Malfatti et al. observed a reduced yield in the French cohort of NMD patients (13.79%), likely reflecting prior extensive diagnostic evaluation before WES testing [14]. Furthermore, panel-based whole-exome sequencing demonstrated a 19% diagnostic yield in a large multicentric heterogeneous cohort of neuromuscular disorders, with many presenting non-specifically [9].

Nearly all disease groups in our study demonstrated high diagnostic yield, while a group of UND had a markedly lower yield (Figure 1), suggesting greater genetic heterogeneity or lower detectability within that subgroup. Our findings suggest that NGS performance is not uniform across neuromuscular phenotypes. This reflects differences in the underlying genetic architecture, phenotypic heterogeneity, or current limitations of testing strategies for nonspecific neuromuscular presentations. In a study of targeted gene panel usefulness in 75 adolescents and adults with asymptomatic or minimally symptomatic hyperCKaemia, conclusive genetic diagnosis was achieved in 24% of cases [15]. This relatively modest diagnostic yield supports the notion that clinically nonspecific neuromuscular presentations, such as isolated hyperCKaemia, are associated with a lower likelihood of achieving a definitive molecular diagnosis. In our cohort, the majority of patients within the unclassified neuromuscular disease (UND) group presented with hyperCKaemia, which likely explains the similarly reduced diagnostic yield observed in this subgroup. Moreover, our findings suggest that, although certain phenotypes such as hyperCKaemia numerically predominate within the UND group, the likelihood of achieving a molecular diagnosis by NGS does not significantly differ among less specific clinical presentations. In other words, within the UND subset of patients, clinical phenotype alone may have limited discriminatory value in predicting diagnostic success.

Our findings align well with those by Chen et al., who reported a relatively high WES diagnostic yield (63.4%) in neuromuscular disorders when sequencing was employed as a second-tier test after an initial diagnostic workup [16]. Notably, their cohort consisted predominantly of adult-onset patients, suggesting that the observed high yield was not population-specific but rather reflects the impact of appropriate clinical preselection.

High diagnostic yield was also reported in pediatric neuromuscular disorders within a consanguineous population, a setting typically associated with increased detection rates due to recessive inheritance [17]. The diagnostic approach in the aforementioned study was based on next-generation sequencing following detailed phenotypic evaluation, therefore resembling our own. Despite differences in population structure and geographic origin, the similarly high yield observed in both studies underscores the role of phenotype-driven selection of patients in optimizing genetic testing. Overall, the diagnostic yield appears to decline in cohorts characterized by greater clinical heterogeneity and extensive prior genetic work-up. On the other hand, the diagnostic performance of WES seems strongly influenced by prior phenotypic characterization and patient selection [16].

In our study, WES enabled the identification of diverse and unexpected genetic etiologies in patients with suspected NMDs, including extremely rare PURA syndrome and even copy number variants (later confirmed by molecular karyotyping) confirming a case of Prader–Willi syndrome. These findings highlight the broad phenotypic spectrum that may mimic primary myopathies and underscore the value of comprehensive genomic analysis in differentiating primary myopathies from syndromic or acquired conditions. In several cases in our cohort, histopathological or electrophysiological findings initially suggested a primary myopathic process, emphasizing the importance of molecular confirmation. Our findings, therefore, underscore the critical role of detailed phenotypic classification in neuromuscular genetics. When patients could be assigned to established disease categories, genetic testing demonstrated near-complete diagnostic yield.

The diagnostic yield of neuromuscular genetic testing continues to improve with the integration of multi-omics approaches. Namely, Marchant et al. showed that combining exome sequencing with genome and RNA analyses nearly doubled the diagnostic rate (from 34% to 62%) in unsolved cases [18]. In a large whole-genome sequencing study of rare genetic disorders in 1452 Korean families, neuromuscular disorders had the highest diagnostic yield among all disease categories (62.4%) [19]. This finding confirms that neuromuscular phenotypes are particularly amenable to genetic diagnosis, especially when comprehensive genomic approaches are applied. We can assume that future diagnostic workflows for NMDs will increasingly rely on layered genomic and functional analyses, particularly for patients with nonspecific or unresolved phenotypes. Although no initially negative cases were reclassified as positive at the time of study completion, ongoing longitudinal reanalysis is an integral component of contemporary genomic diagnostics and has the potential to improve diagnostic yield as knowledge and bioinformatic approaches evolve.

Additionally, in our cohort, nonsense variants accounted for 19.6% of all detected mutations, which is higher than the ~11% proportion of disease-causing nonsense mutations reported in the general genetic disease literature, suggesting a relative enrichment of truncating variants in a population of patients with NMDs [20]. However, this finding should be interpreted with caution, as patients with the most frequent genetic findings in SMA, DMD and myotonic dystrophy were excluded from this analysis.

Several limitations of our study should be acknowledged, including the single-center design and incomplete availability of electrophysiological and histopathological data in a subset of patients. The exclusion of patients with genetically confirmed DMD/BMD and SMA, who are typically diagnosed by targeted testing, may have resulted in a relative enrichment of diagnostically unresolved cases, potentially leading to an overestimation of the observed NGS diagnostic yield. Although the regression model met general assumptions for multivariable analysis, the sample size may have limited statistical power to detect modest associations with a positive WES diagnostic outcome, and this should be considered when interpreting non-significant findings.

The strengths of our study include a relatively large patient cohort and the presentation of a decade-long experience of a specialized center for the evaluation and treatment of neuromuscular disorders. This is the first study assessing the contribution of WES in pediatric patients with NMDs in Serbia.

## 4. Materials and Methods

### 4.1. Patients

Our investigation was a combined retrospective–prospective cohort study of patients who underwent NGS-based diagnostics due to suspicion of pediatric-onset neuromuscular disorders (NMDs) (age < 18 years). These pediatric patients were referred for evaluation to the Mother and Child Health Care Institute of Serbia “Dr Vukan Čupić” over a ten-year period, from 2015 to 2025.

Importantly, to align with standard diagnostic algorithms, we applied specific inclusion criteria for patients presenting with clinical phenotypes strongly suggestive of Duchenne/Becker muscular dystrophy (DMD/BMD) or spinal muscular atrophy (SMA). Such patients were included in the study exclusively if prior first-tier targeted genetic testing—more specifically, screening for copy number variations (deletions and duplications) in the DMD gene and the typical homozygous deletion of exon 7 in the SMN1 gene—yielded negative results. Therefore, fifty-three patients with DMD/BMD and 16 patients with SMA were not included in this study. Furthermore, 27 patients diagnosed with congenital and childhood-onset forms of myotonic dystrophy type 1 were excluded from the study cohort. These specific exclusions were applied because standard short-read NGS methodologies have well-documented limitations in reliably detecting large copy number variations (e.g., in DMD and SMN1) and nucleotide repeat expansions (characteristic of myotonic dystrophy), for which targeted molecular assays remain the gold standard.

Initial clinical suspicion of an underlying neuromuscular disorder was primarily driven by hypotonia accompanied by focal or generalized muscle weakness, alongside distinct physical findings (e.g., myopathic face and calf pseudohypertrophy) and biochemical markers such as elevated serum creatine kinase. Based on these dominant clinical phenotypes prior to genetic testing, the cohort was stratified into seven specific diagnostic categories: (1) congenital myopathies (CMs) and congenital muscular dystrophies (CMDs); (2) limb-girdle muscular dystrophies (LGMDs); (3) Duchenne/Becker muscular dystrophies exclusively with point mutations in the DMD gene; (4) congenital myasthenic syndromes (CMSs); (5) neuropathies and lower motor neuron lesions (excluding spinal muscular atrophy with homozygous deletion of exon 7 in the SMN1 gene); (6) metabolic myopathies; and (7) unclassified neuromuscular diseases (UNDs).

Demonstrating the highest degree of clinical heterogeneity, the UND group consisted of children whose clinical presentation did not suggest a distinct neuromuscular disorder. In addition to muscle weakness, these patients often exhibited dysmorphism and/or multi-organ involvement. This group also included patients with rhabdomyolysis of unknown etiology or isolated, asymptomatic hyperCKemia.

Data were collected from patients’ medical histories and during outpatient examinations. The following data were collected: patient age, sex, age at onset of the first symptoms, age at genetic diagnosis, signs and symptoms of the disease, comorbidities, family history, and available findings of key diagnostic procedures performed (electroneuromyography, muscle biopsy, cardiological findings, genetic tests, and metabolic analyses). Phenotypic characteristics of the disease were classified according to the Human Phenotype Ontology (HPO) nomenclature [21].

### 4.2. Ethics Statement

The study was approved by the Institutional Ethics Committee of the Mother and Child Health Care Institute of Serbia (protocol code 8/112, approval date: 25 September 2025). The research was conducted in accordance with the principles of the Declaration of Helsinki. Informed consent for genetic testing and diagnostic procedures was obtained from parents and legal guardians. All genetic and diagnostic procedures were performed as part of routine clinical practice and not specifically for this study.

### 4.3. Exome Sequencing, Data Analysis and Interpretation

Whole-exome sequencing was applied as an NGS diagnostic modality in a vast majority of patients in our cohort (84%). Genomic DNA was isolated from whole-blood samples of the patients and relevant family members using the QIAamp DNA-Blood-Mini-Kit (QIAGEN, Hilden, Germany) and subsequently analyzed by Next-Generation Sequencing (NGS) using Clinical Exome Sequencing (CES) or Whole-Exome Sequencing (WES). CES was performed using Illumina DNA Prep with the Enrichment protocol, with the TruSight One Gene Panel comprising 4813 known disease-associated genes (Illumina, San Diego, CA, USA), while WES was performed using DNA Prep with the Exome 2.5 Enrichment protocol (Illumina, San Diego, CA, USA). Sequencing (with 150 bp paired-end runs) was performed on the Illumina NextSeq 2000 System (Illumina, San Diego, CA, USA), with alignment and analysis performed against the GRCh38/hg38 reference genome assembly. Variants passing quality call (QC) filters (quality score > Q20, read depth > 20, and percentage of variant frequency for the minor allele > 20%) and with minor allele frequencies < 1% in The Genome Aggregation Database (gnomAD) were further analyzed. Systematic interpretation of variants was performed using VarSome Clinical (Saphetor, Lausanne, Switzerland) and the ClinVar database [22,23]. All variants detected were classified using American College of Medical Genetics and Genomics (ACMG) guidelines [24,25]. Mendeliome-based NGS analysis was performed in 11%, while a targeted neuromuscular gene panel was applied in 5% of patients. Subsequent parental segregation analysis was done in case of a detected causative or potentially causative variant. Likely pathogenic variants and VUSs were considered relevant for the genetic diagnosis of neuromuscular disorders if clinical findings and the results of segregation analysis supported their significance in confirming the diagnosis. At the time of data analysis for this study, no updates indicating conversion from negative findings to positive findings had been reported for any of the initially undiagnosed patients.

## 5. Conclusions

Our findings support the implementation of an early, phenotype-driven, genomics-first approach in the diagnostic evaluation of pediatric neuromuscular disorders. Exome sequencing substantially improves diagnostic accuracy and efficiency, reduces the need for invasive procedures, facilitates timely treatment initiation in certain cases, and enables appropriate genetic counseling. Paradoxically, our findings suggest that the success of advanced genomic technologies does not diminish the role of clinical expertise but rather depends on it—precision in diagnosis begins not with sequencing but with the clinician’s ability to recognize the phenotype. Patients with less specific neuromuscular presentations continue to represent a major diagnostic challenge, despite the availability of advanced NGS technologies.

## Figures and Tables

**Table 1:** Age of onset.

Age of Onset	%
At birth	27.0
≤12 months	24.0
12–36 months	13.0
3–6 years	10.0
6–12 years	15.0
12–18 years	9.0

**Table 2:** Distribution of patients by suspected clinical diagnostic category prior to genetic testing.

Diagnostic Category	Percentage (%)
Unclassified neuromuscular diseases	34.0
Duchenne/Becker muscular dystrophies	19.0
Limb-girdle muscular dystrophies	17.0
Neuropathies and lower motor neuron lesions	10.0
Congenital myopathies and dystrophies	8.0
Metabolic myopathies	8.0
Congenital myasthenic syndromes	4.0
Total	100.0

**Table 3:** Frequency distribution of genes with identified mutations in pediatric patients with suspected neuromuscular disorders using NGS methods.

Gene	Frequency (*n*)
*DMD*	17
*RYR1*	6
*LAMA2*	5
*TTN* and *PMP22*	3
*GAA*, *MYH2. CHRNE*, *ORAI1*, *MT-ATP6*, and *IGHMBP2*	2
*DOK7*, *POMT1*, *POMT2*, *IFT140*, *CAPN3*, *RPGRIP1*, *RAB2B*, *MECP2*, *PLP1*, *PURA*, *COL1A2*, *COL6A1*, *CACNA1A*, *HINT1*, *AP4E1*, *SGCG*, *CHAT*, *SCN4A*, *ALDH5A1*, *TARS2*, *TYMP*, *PLA2G6*, *LMNA*, *ERF*, and *NDRG1*	1

**Table 4:** Multivariable logistic regression analysis of predictors of a positive WES diagnostic yield.

Variable	OR (Exp(B))	95% CI	*p*-Value
Sex	0.69	0.23–2.05	0.500
Disease group	0.64	0.49–0.83	0.001
Age at onset	1.10	0.81–1.49	0.541
Family history	0.76	0.36–1.64	0.489
Comorbidity	1.76	0.91–3.41	0.092

OR—odds ratio; CI—confidence interval.

## Data Availability

The data presented in this study are available upon request from the corresponding author due to legal or ethical reasons.

## References

[1] Rathore G., Kang P.B. (2023). Pediatric neuromuscular diseases. Pediatr. Neurol..

[2] Woodcock I.R., Fraser L., Norman P., Pysden K., Manning S., Childs A.M. (2016). The prevalence of neuromuscular disease in the paediatric population in Yorkshire, UK: Variation by ethnicity and deprivation status. Dev. Med. Child Neurol..

[3] Norwood F.L., Harling C., Chinnery P.F., Eagle M., Bushby K., Straub V. (2009). Prevalence of genetic muscle disease in Northern England: In-depth analysis of a muscle clinic population. Brain.

[4] Piñeros-Fernández M.C., Morte B., García-Giménez J.L. (2024). Utility of exome sequencing for the diagnosis of pediatric-onset neuromuscular diseases beyond diagnostic yield: A narrative review. Neurol. Sci..

[5] Barbosa-Gouveia S., Vázquez-Mosquera M.E., González-Vioque E., Hermida-Ameijeiras Á., Sánchez-Pintos P., de Castro M.J., León S.R., Gil-Fournier B., Domínguez-González C., Camacho-Salas A. (2022). Rapid molecular diagnosis of genetically inherited neuromuscular disorders using next-generation sequencing technologies. J. Clin. Med..

[6] Ng K.W.P., Chin H.L., Chin A.X.Y., Goh D.L. (2022). Using gene panels in the diagnosis of neuromuscular disorders: A mini-review. Front. Neurol..

[7] Kulsirichawaroj P., Chanvanichtrakool M., Wattanadilokchatkun P., Pho-Iam T., Limwongse C., Likasitwattanakul S., Boonyapisit K., Sanmaneechai O., Nishino I., Shotelersuk V. (2025). Next-generation sequencing for pediatric-onset neuromuscular disorders unresolved by conventional diagnostic methods. Pediatr. Res..

[8] Tsang M.H.Y., Chiu A.T.G., Kwong B.M.H., Liang R., Yu M.H.C., Yeung K.S., Ho W.H.L., Mak C.C.Y., Leung G.K.C., Pei S.L.C. (2020). Diagnostic value of whole-exome sequencing in Chinese pediatric-onset neuromuscular patients. Mol. Genet. Genom. Med..

[9] Westra D., Schouten M.I., Stunnenberg B.C., Kusters B., Saris C.G.J., Erasmus C.E., Van Engelen B.G., Bulk S., Verschuuren-Bemelmans C.C., Gerkes E.H. (2019). Panel-based exome sequencing for neuromuscular disorders as a diagnostic service. J. Neuromuscul. Dis..

[10] Herman I., Lopez M.A., Marafi D., Pehlivan D., Calame D.G., Abid F., Lotze T.E. (2021). Clinical exome sequencing in the diagnosis of pediatric neuromuscular disease. Muscle Nerve.

[11] Fattahi Z., Kalhor Z., Fadaee M., Vazehan R., Parsimehr E., Abolhassani A., Beheshtian M., Zamani G., Nafissi S., Nilipour Y. (2017). Improved diagnostic yield of neuromuscular disorders applying clinical exome sequencing. Clin. Genet..

[12] Lee H.F., Chi C.S., Tsai C.R. (2021). Diagnostic yield and treatment impact of whole-genome sequencing in paediatric neurological disorders. Dev. Med. Child Neurol..

[13] Babić Božović I., Maver A., Leonardis L., Meznaric M., Osredkar D., Peterlin B. (2021). Diagnostic yield of exome sequencing in myopathies. PLoS ONE.

[14] Malfatti E., Caramizaru A., Lee H., Kim J., Shoaito H., Pennisi A., Souvannanorath S., Authier F.J., Dumitrescu A., Fahmy N. (2025). NEUROMYODredger: Whole exome sequencing for neuromuscular disorders. Clin. Genet..

[15] Gemelli C., Traverso M., Trevisan L., Fabbri S., Scarsi E., Carlini B., Prada V., Mongini T., Ruggiero L., Patrone S. (2022). Integrated approach to hyperCKemia evaluation. Muscle Nerve.

[16] Chen P.S., Chao C.C., Tsai L.K., Huang H.Y., Chien Y.H., Huang P.H., Hwu W.L., Hsieh S.T., Lee N.C., Hsueh H.W. (2023). Diagnostic challenges after WES in neuromuscular disorders. J. Neuromuscul. Dis..

[17] Al-Hedaithy A., Alghamdi F., Almomen M., Amer F., Al Dossari S., Noreen Baig D., Bashir S. (2025). Genetic diagnostic evaluation in pediatric neuromuscular diseases. Sci. Rep..

[18] Marchant R.G., Bryen S.J., Bahlo M., Cairns A., Chao K.R., Corbett A., Davis M.R., Ganesh V.S., Ghaoui R., Jones K.J. (2024). Genome and RNA sequencing boost neuromuscular diagnoses to 62% from 34% with exome sequencing alone. Ann. Clin. Transl. Neurol..

[19] Lee S., Seo G.H., Kim S.Y., Jang S.S., Jang S., Choi S., Chin H., Lee S.J., Oh D.E., Ryu S.W. (2025). Clinical utility of genome sequencing in rare diseases: Lessons from a single-center study of 1,452 Korean families. npj Genom. Med..

[20] Temaj G., Telkoparan-Akillilar P., Nuhii N., Chichiarelli S., Saha S., Saso L. (2023). Recoding of Nonsense Mutation as a Pharmacological Strategy. Biomedicines.

[21] Gargano M.A., Matentzoglu N., Coleman B., Addo-Lartey E.B., Anagnostopoulos A.V., Anderton J., Avillach P., Bagley A.M., Bakštein E., Balhoff J.P. (2024). The Human Phenotype Ontology in 2024: Phenotypes around the world. Nucleic Acids Res..

[22] Landrum M.J., Lee J.M., Riley G.R., Jang W., Rubinstein W.S., Church D.M., Maglott D.R. (2014). ClinVar: Public archive of relationships among sequence variation and human phenotype. Nucleic Acids Res..

[23] Kopanos C., Tsiolkas V., Kouris A., Chapple C.E., Albarca Aguilera M., Meyer R., Massouras A. (2019). VarSome: The human genomic variant search engine. Bioinformatics.

[24] Richards S., Aziz N., Bale S., Bick D., Das S., Gastier-Foster J., Grody W.W., Hegde M., Lyon E., Spector E. (2015). Standards and guidelines for the interpretation of sequence variants. Genet. Med..

[25] Durkie M., Cassidy E.J., Berry I., Owens M., Turnbull C., Scott R.H., Taylor R.W., Deans Z.C., Ellard S., Baple E.L. (2024). ACGS Best Practice Guidelines for Variant Classification in Rare Disease 2024.

